# Beneficial Effects of Fermented Blueberry Pomace Supplementation on Carcass Traits, Meat Quality, and Antioxidant Capacity of Spent Hens

**DOI:** 10.3390/ani15192799

**Published:** 2025-09-25

**Authors:** Binghua Qin, Ting Chen, Zhihua Li, Wei Lan, Yadong Cui, Md. Abul Kalam Azad, Xiangfeng Kong

**Affiliations:** 1 Hunan Provincial Key Laboratory of Animal Nutritional Physiology and Metabolic Process, National Engineering Laboratory for Pollution Control and Waste Utilization in Livestock and Poultry Production, Institute of Subtropical Agriculture, Chinese Academy of Sciences, Changsha 410125, China; qinbinghua23@mails.ucas.ac.cn (B.Q.); chenting23@mails.ucas.ac.cn (T.C.); zhli92@126.com (Z.L.); 2 School of Biology and Food Engineering, Fuyang Normal University, Fuyang 236037, China; lanwei@fynu.edu.cn (W.L.); fysyswx@163.com (Y.C.); 3 College of Advanced Agricultural Sciences, University of Chinese Academy of Sciences, Beijing 100049, China

**Keywords:** fermented blueberry pomace, meat quality, molecular docking, network pharmacology, spent hen

## Abstract

This study aimed to evaluate the effects of fermented blueberry pomace (FBP) supplementation on carcass traits, meat quality, and antioxidant capacity in spent hens and further investigated underlying mechanisms using network pharmacology and molecular docking analyses. A total of 320 Yukou Jingfen No. 8 spent hens (345 days old) were fed a basal diet supplemented with 0, 0.25, 0.5, or 1.0% FBP for 56 days. Each group contained eight replicates with ten hens per replicate. Results showed that 0.25% FBP reduced the percentage of thigh muscle, whereas 0.5% FBP reduced drip loss at 24 h post-mortem in the breast muscle compared to the control group (*p* < 0.05). Additionally, 0.5% FBP enhanced the total antioxidant capacity compared to the 0.25% group and increased superoxide dismutase activity compared to the control and 0.25% FBP groups (*p* < 0.05). In the breast muscle, 0.25 and 0.5% FBP reduced drip loss compared to the control group at 48 h post-mortem, while 0.25% FBP upregulated glutathione peroxidase 1 expression more than the other groups (*p* < 0.05). Network pharmacology analysis identified 302 targets related to the potential bioactive compounds in FBP and 401 targets associated with meat quality, such as core targets of insulin, protein kinase cAMP-activated catalytic subunit beta, steroid receptor coactivator, etc. The identified key signaling pathways included JAK-STAT and PI3K-Akt signaling pathways, which were related to protein synthesis and muscle growth. Molecular docking analysis confirmed strong binding activity between bioactive compounds of FBP and meat quality. In conclusion, dietary FBP supplementation enhances the breast muscle quality of spent hens through the potential bioactive compounds targeting insulin and modulating JAK-STAT and PI3K-Akt signaling pathways, without affecting carcass traits. Moreover, 0.5% FBP exhibited better effects than other treatment groups in spent hens.

## Simple Summary

Blueberry pomace is rich in bioactive compounds, such as anthocyanins and polyphenols, which have exhibited antioxidant and anti-inflammatory properties. This study evaluated the effects of fermented blueberry pomace (FBP) supplementation on carcass traits, meat quality, and antioxidant capacity in spent hens. Additionally, we investigated the underlying mechanisms of these effects using network pharmacology and molecular docking analyses. The findings indicated that dietary FBP supplementation enhances the meat quality of spent hens through the potential bioactive compounds targeting their regulatory pathways without affecting carcass traits. This study also provides the pioneering application of FBP in layer diets, coupled with the adoption of the network pharmacology approach to systematically predict the multi-component, multi-target, and multi-pathway mechanisms by which FBP improved meat quality. Together, this approach establishes a novel theoretical basis for addressing waste valorization and enhancing the quality of livestock products simultaneously.

## 1. Introduction

The Yukou Jingfen No. 8 layer, commonly known as the black phoenix layer, is an excellent crossbred known for its better local environmental adaptability. This breed also has the advantages of a long peak laying period and low production costs. However, layers often face culling from the layer industry due to a rapid decline in the production and quality of eggs during the late laying phase. Despite this, the meat of these spent hens is rich in protein, essential fatty acids, vitamins, minerals, and various umami amino acids, thereby providing it with high nutritional value [1]. However, the enlarged diameter and reduced density of muscle fibers, combined with the elevated insoluble collagen content in the muscle of spent hens, lead to a reduced water-binding capacity, diminished tenderness, and poor overall meat quality [2]. These factors contribute to lower consumer demand. Moreover, meat from spent hens is more prone to oxidative deterioration during processing and storage than broiler meat, as it has higher concentrations of fatty acids and catalytic metals. Oxidative reactions are one of the significant factors in the quality deterioration of meat products, as both lipid and protein oxidation contribute to the low quality of meat from spent hens [3,4]. Therefore, there is growing interest in improving the meat quality of spent hens using safe and effective nutritional strategies through natural antioxidants, such as phenolic extracts, spices, Chinese herbs, berries, and vitamins [5,6].

Blueberry, a fruit originating from North America, is mainly distributed in the Guizhou, Sichuan, and Anhui provinces of China. It is rich in organic acids, phenolic compounds, and other bioactive compounds, and has exhibited various health benefits, including antioxidant and anti-inflammatory properties [7]. A previous study reported that blueberry polyphenol extract supplementation increased catalase (CAT) and superoxide dismutase (SOD) activities in the jejunum and ileum of weaned rats by modulating the Nrf2-Keap1 signaling pathway [8]. Additionally, blueberry proanthocyanidins were found to decrease interleukin-6 (IL-6) and tumor necrosis factor-α (TNF-α) levels in mice through the NF-κB signaling pathway [9]. Given these characteristics, blueberry extracts have gained increasing attention as natural feed additives to enhance product quality in livestock and poultry production. After processing, large amounts of blueberry pomace remain unused and have not been effectively utilized because of their high moisture content and anti-nutritional factors. Microbial fermentation with excipients can reduce the moisture content, ultimately solving storage and transportation problems. Simultaneously, this process enhances probiotic activity and degrades anti-nutritional factors, thereby improving the nutritional value of the pomace. Previous studies have indicated that microbial fermentation can enhance the nutritional value of the blueberry pomace [10]. At present, fermented blueberry pomace (FBP) is mainly explored for its potential to reduce obesity and alleviate inflammation in mice fed a high-fat diet [11,12]. Our previous study indicated that diets supplemented with FBP not only enhance the quality and nutritional value of eggs during the late-phase laying period by enhancing ovarian antioxidant capacity and liver lipid metabolism, but also improve intestinal health by upregulating *mucin 2* expression and anti-inflammatory factors, and increasing the abundance of beneficial bacteria [13,14]. However, the effects of dietary FBP on carcass traits, meat quality, and antioxidant capacity in spent hens have not yet been explored.

Network pharmacology has rapidly advanced in recent years, providing innovative research approaches and methodologies for both traditional Chinese medicine and modern medical applications [15]. The analytical methodology of network pharmacology primarily encompasses the following steps: The process typically begins with screening active pharmaceutical ingredients and their potential targets through pharmacological databases. Subsequently, disease-associated targets are collected to identify the drug-disease intersection targets, which represent the potential therapeutic targets. These intersection targets are then utilized to construct a protein–protein interaction (PPI) network, followed by conducting functional enrichment analysis using Gene Ontology (GO) and pathway enrichment analysis using the Kyoto Encyclopedia of Genes and Genomes (KEGG). A multi-dimensional “compound–target–pathway” network is established to systematically predict and elucidate the multi-component, multi-target, and multi-pathway mechanisms underlying traditional Chinese medicine [16]. Additionally, network pharmacology is also a systematic technique to evaluate the underlying mechanisms and functions of natural bioactive components in supplements, allowing for the prediction of multiple targets and signaling pathways associated with various conditions.

Considering the aforementioned advantages of FBP, we hypothesized that dietary FBP supplementation could enhance carcass traits, meat quality, and antioxidant capacity of spent hens. Thus, to test the hypothesis, this study investigated the effects of dietary FBP on carcass traits, meat quality, and the antioxidant capacity of breast muscles in spent hens. Furthermore, it sought to explore the mechanisms by which FBP influences meat quality using network pharmacology and molecular docking analyses. The objective was to provide a scientific foundation for improving the meat quality of spent hens through nutritional strategies and to maximize the utilization of fruit by-products.

## 2. Materials and Methods

### 2.1. Preparation of FBP

The fresh blueberry pomace was obtained from Anhui Xiuqin Agricultural Technology Co., Ltd. (Fuyang, China). The preparation and nutritional composition of FBP were consistent with our previous study [13]. Briefly, the FBP was prepared by fermenting blueberry pomace with a combined microbial starter, including microbial agent (*Baclillus subtilis*, *Bacillus licheniformis*, *Lactobacillus plantarum*, and marine yeast), bioactive enzyme, culture medium 1 (ginseng leaves), culture medium 2 (soybean powder), and sugar for 64 h under the conditions of a temperature of 75 °C, a relative humidity of 45% to 55%, and a dissolved oxygen content of 3% to 10%.

### 2.2. Animals and Feeding Management

A total of 320 healthy Yukou Jingfen No. 8 layers (345 days old) were randomly selected to conduct a feeding experiment at the commercial layer farm located in Fuyang, China. The selected layers were allocated into four groups, and each group consisted of eight replicates, with ten hens per replicate. Based on our previous study [13], experimental layers were fed a basal diet supplemented with 0, 0.25%, 0.5%, or 1.0% FBP for 56 days. All hens were reared in wire cages with five hens per cage, had free access to food and water at all times, and were monitored regularly. The hens were housed in a controlled temperature room at 18–23 °C with a relative humidity of 55–65%, and a lighting of 16/8 h light/dark cycle. The basal diet was formulated according to the China National Feeding Standard of Chicken (NY/T 33-2004) [17], and the composition and nutrient levels of the basal diet are shown in Table 1.

### 2.3. Sample Collection

On day 56 of the feeding trial, one bird from each replicate (*n* = 8) was randomly selected for sampling. Upon collecting the body weight of each selected bird, the birds were euthanized by CO_2_ asphyxiation and cervical dislocation. The left breast (middle position) and thigh (mid-point) muscles were collected for meat quality analysis. Additionally, one part of the breast muscle was immediately kept in snap-frozen liquid nitrogen and stored at −80 °C to analyze oxidative stress status and gene expression.

### 2.4. Carcass Traits and Organ Index Analysis

Carcass traits (including percentages of breast and thigh muscles, eviscerated yield, and half-eviscerated yield) and organ index (including the liver, spleen, and abdominal fat) were calculated based on body weight, carcass weight, and eviscerated weight recorded during slaughter. These calculations followed the guidelines outlined in Performance Terminology and Measurements for Poultry (NY/T 823-2020) [18].

### 2.5. Meat Quality Analysis

A pH Star meter (Matthaus, Berlin, Germany) was used to measure the pH values of the breast and thigh muscles at 45 min (pH_45min_) and 48 h (pH_48h_) post-mortem. A CR-410 colorimeter (Kinica Minolta Sensing Inc., Shanghai, China) was used to measure the meat color indicators of the breast and thigh muscles at 45 min, including lightness (L*), redness (a*), and yellowness (b*) values. Meat quality indicators, including drip loss and cooking loss of the breast and thigh muscles, were determined in accordance with the standard guidelines of the Determination of Livestock and Poultry Meat Quality (NY/T 1333-2007) [19]. Briefly, 10 g of muscle sample was weighed (w_1_) and placed in a drip loss tube at 4 °C. The sample was taken out after 24 and 48 h, respectively, and weighed (w_2_ and w_3_) after wiping the surface moisture. The drip loss was calculated according to the following formulas: 24 h drip loss (%) = (w_1_ − w_2_)/w_1_ × 100 and 48 h drip loss (%) = (w_1_ − w_3_)/w_1_ × 100. To determine the cooking loss, approximately 10 g of muscle sample was weighed (w_4_) and wrapped in aluminum foil. After boiling for 30 min, the sample was cooled and weighed (w_5_). The cooking loss was calculated as follows: cooking loss (%) = (w_4_ − w_5_)/w_4_ × 100. After determining the cooking loss, the muscle samples were trimmed into small strips. The strips were then used to determine shear force (N) using a GR-150 tenderness meter (G & R General Corporation, Shakopee, MN, USA).

### 2.6. Oxidative Stress Status Analysis

The breast muscle samples (approximately 100 mg) preserved at −80 °C were thawed naturally and quickly homogenized with ice-cold physiological saline at 1:9 (*w*/*v*). Afterward, the samples were centrifuged for 10 min at 2000× *g* to collect the supernatants. The supernatants were further used to assess oxidative status indicators using the commercial assay kits (Nanjing Jiancheng Biotechnology Institute, Nanjing, China). The oxidative status indicators included the activities of glutathione (GSH), GSH peroxidase (GSH-PX), SOD, and total antioxidant capability (T-AOC), as well as the concentration of malondialdehyde (MDA). Measurements were performed on a Microplate Reader (Infinite M200 PRO; Tecan, Männedorf, Switzerland) based on the instructions provided by the manufacturer.

### 2.7. Gene Expression Analysis

The total RNA of the naturally thawed breast muscle sample (100 mg) was extracted with AG RNAex Pro reagent (Accurate Biology, Changsha, China) according to the manufacturer’s instructions. The purity and concentration of the extracted RNA were determined by a NanoDrop ND-2000 spectrophotometer (Thermo Fisher Scientific, Waltham, MA, USA). Then, the extracted RNA was reverse-transcribed into cDNA using an Evo M-MLV RT kit (Accurate Biology, Changsha, China). The real-time quantitative PCR (RT-qPCR) analysis was performed on a LightCycler^®^ 480II Real-Time PCR System (Roche, Basel, Switzerland) with the SYBR^®^ Green Premix Pro Taq HS qPCR Kit (Accurate Biology, Changsha, China). The RT-qPCR was carried out with a 10 µL reaction volume, which included 1 µL of cDNA, 0.4 µL of forward primer, 0.4 µL of reverse primer, 5 µL of SYBR^®^ Green Premix (Accurate Biology, Changsha, China), and ddH_2_O up to 10 µL. After a cycle of pre-denaturation at 95 °C for 5 min, 45 cycles were performed, including denaturation at 95 °C for 10 s, annealing at 53 °C for 10 s, and extension at 72 °C for 20 s. Finally, an extension was carried out at 72 °C for 5 min. The primer sequences are presented in Table 2. The relative mRNA expression for each gene was normalized to *β-actin* using the 2^−ΔΔCt^ method [20].

### 2.8. Network Pharmacology Analysis

Bioactive compounds of FBP were sourced from the China National Knowledge Infrastructure (https://www.cnki.net/; accessed on 6 January 2025) and PubMed (https://pubmed.ncbi.nlm.nih.gov/; accessed on 6 January 2025) databases. Subsequently, the Swiss Target Prediction (http://www.swisstargetprediction.ch/; accessed on 6 January 2025) database was employed to identify potential targets of FBP with a probability value ≥ 0. The potential targets of FBP related to the meat quality of layers were identified by searching the National Center for Biotechnology Information (https://www.ncbi.nlm.nih.gov/; accessed on 6 January 2025) database using the keywords “meat”, “muscle”, and “gallus”. The intersection targets between the meat quality-related genes and predicted FBP targets were determined. A Venn diagram was then constructed using GenesCloud (https://www.genescloud.cn/; accessed on 6 January 2025) and Cytoscape 3.7.1 software to visualize the compound-target network.

The intersecting gene targets were imported into the STRING database (https://cn.string-db.org; accessed on 8 January 2025). The PPI network and TSV format file were obtained after setting the species to “*Gallus*” and using the confidence level ≥ 0.7. The TSV format files were then imported into Cytoscape 3.7.1 for visualization. Core targets were identified using the betweenness centrality (BC) parameters in the Cyto NCA plug-in, and a new PPI network was constructed.

GO enrichment and KEGG pathway enrichment analyses data were collected using the Database for Annotation, Visualization, and Integrated Discovery (https://davidbioinformatics.nih.gov/; accessed on 8 January 2025). The biological processes (BP), cellular components (CC), and molecular functions (MF) of intersection targets were identified using the GO enrichment analysis. Additionally, key signaling pathways were identified using the KEGG pathway enrichment analysis.

### 2.9. Molecular Docking

Based on the BC values, the top four core targets associated with meat quality within the PPI network were selected for molecular docking with the bioactive compounds of FBP. Subsequently, the potential interactions between the bioactive compounds and the core targets were assessed. Briefly, the molecular docking processes were conducted as follows: Firstly, the structure of the bioactive compound was acquired from the TCMSP database (https://old.tcmsp-e.com/tcmsp.php; accessed on 13 January 2025), converted into a 3D structure using the PubChem database (https://pubchem.ncbi.nlm.nih.gov/; accessed on 13 January 2025), and then saved in PDB format using OpenBabel 3.1.1 software. Secondly, the target structure was retrieved from the RCSB-PDB database (https://www.rcsb.org/; accessed on 13 January 2025), afterward subjected to dehydration and hydrogenation processes using PyMOL 2.6.2 software and saved as a PDBQT file. Finally, molecular docking was performed using Autodock 1.5.7 software to calculate the minimal binding energy. The results were visualized using PyMOL 2.6.2 software.

### 2.10. Statistical Analyses

The data are presented as means with their standard error of the means (SEM). Data normality was assessed using the Shapiro–Wilk test, and homogeneity of variances was verified using Levene’s test as part of the one-way ANOVA procedure. For datasets that met both assumptions of normality and homogeneity of variances, differences were analyzed by one-way ANOVA followed by Tukey’s post-hoc test. For other datasets, the Kruskal–Wallis test was used for nonparametric analysis. To analyze the effects of FBP supplementation in spent hens’ diets, linear and best-fit quadratic models were determined using the regression curves. All statistical analyses were performed using IBM SPSS version 26.0 (IBM Inc., Chicago, IL, USA). Results were considered statistically significant at *p* < 0.05, and a trend toward significance was considered for 0.05 ≤ *p* < 0.10. Throughout group allocation, the experimental trial, laboratory analyses, and data analysis, only Binghua Qin and Xiangfeng Kong were aware of the group allocation.

The mathematical model for one-way ANOVA used in the present study was as follows:

(1) $$Y_{ij}=\mu +{ɑ}_{i}+\beta_{j}+{ɑ\beta }_{ij}+\varepsilon_{ij}$$

where *Y*_ij_ is the dependent variable, *μ* is the overall mean, *α*_i_ is the fixed treatment effect, *β*_j_ is the day effect, *αβ*_ij_ is the interaction effect between treatment and day, and *ε*_ij_ is the error term.

The mathematical model for polynomial regression in the present study was as follows:

(2) $$y=\beta_{0}+\beta_{1}x+\beta_{2}x^{2}+\varepsilon$$

where *y* is the dependent variable, *x* is the independent variable; *β*_0_, *β*_1_, and *β*_2_ are the regression coefficients; and *ε* is the error term.

## 3. Results

### 3.1. Effects of FBP on Carcass Traits and Organ Index of Spent Hens

As presented in Table 3, compared to the control group, diets supplemented with 0.5% FBP exhibited an increasing trend in the percentage of breast muscle (*p* = 0.067) and liver index (*p* = 0.077). In addition, diets supplemented with 0.25% FBP reduced (*p* < 0.05) the percentage of thigh muscle of spent hens compared to the control group. Diets supplemented with FBP had no significant effects on live weight, dressed weight, dressing percentage, percentage of eviscerated yield, and other carcass traits indicators (*p* > 0.05).

### 3.2. Effects of FBP on Meat Quality of Spent Hens

The meat quality of the breast and thigh muscles of spent hens is presented in Table 4. Compared to the control and 0.25% FBP groups, diets supplemented with 0.5% FBP reduced (*p* < 0.05) the drip loss of breast muscle at 24 h post-mortem. At 48 h post-mortem, the drip loss was reduced and cooking loss was increased in the 0.25% and 0.5% FBP groups (*p* < 0.05), while the pH_48h_ value of breast muscle was lower (*p* < 0.05) in all FBP-supplemented groups, regardless of doses, when compared with the control group. Additionally, all FBP-supplemented groups exhibited a decreasing trend in a* value (*p* = 0.079) of thigh muscle regardless of dose compared with the control group. Regression analysis revealed that diets supplemented with FBP had significant linear and quadratic effects on pH_48h_ (*p* = 0.006, quadratic model: Y = 5.903 − 0.813X + 0.643X^2^), drip loss of breast muscle at 24 h (*p* = 0.028, linear model: Y = 6.341 − 3.131X) and 48 h (*p* = 0.044, linear model: Y = 7.091 − 2.456X; *p* = 0.006, quadratic model: Y = 8.296 − 13.444X + 10.659X^2^), and cooking loss (*p* = 0.003, quadratic model: Y = 32.355 + 1.639X − 1.248X^2^) in the breast muscle. However, diets supplemented with FBP had no significant effects on meat color, pH_45min_ of thigh muscle, and other meat quality indicators (*p* > 0.05).

### 3.3. Effects of FBP on the Antioxidant Capacity of Spent Hens

As presented in Table 5, diets supplemented with 0.5% FBP increased (*p* < 0.05) the SOD activity in the breast muscle compared to the control and 0.25% FBP groups. Additionally, T-AOC activity was higher (*p* < 0.05) in the breast muscle of the 0.5% FBP group compared with the 0.25% FBP group, while MDA concentration was higher (*p* < 0.05) in the 0.5% FBP group compared with the control and 1.0% FBP groups. Regression analysis revealed that diets supplemented with FBP had significant quadratic effects on MDA concentration (*p* = 0.015, quadratic model: Y = 0.244 + 0.662X − 0.629X^2^). Diets supplemented with FBP had no significant effects on the activities of GSH and GSH-PX in the breast muscle of spent hens (*p* > 0.05).

### 3.4. Effects of FBP on Oxidative-Status-Related Gene Expressions of Spent Hens

**Figure 1:**
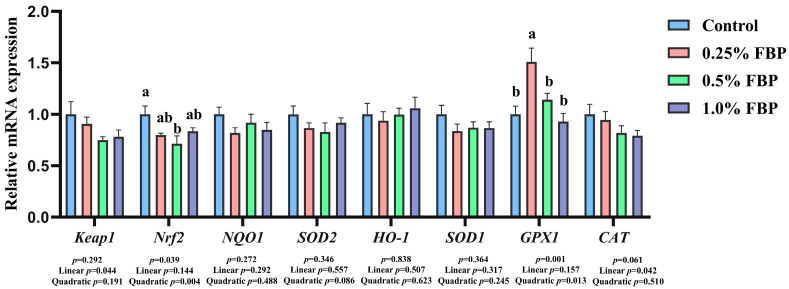
Effects of dietary FBP supplementation on gene expressions related to antioxidant capacity in the breast muscle of spent hens (*n* = 8). ^a–b^ Mean values with different lowercase letter indicate significant difference (*p* < 0.05). *CAT*, catalase; FBP, fermented blueberry pomace; *GPX1*, glutathione peroxidase 1; *HO-1*, heme oxygenase-1; *Keap1*, Kelch-like ECH-associated protein 1; *NQO1*, NAD(P)H quinone dehydrogenase 1; *Nrf2*, nuclear factor erythroid 2-related factor 2; *SOD*, superoxide dismutase.

As presented in Figure 1, the glutathione peroxidase 1 (*GPX1*) expression was upregulated (*p* < 0.05) in the 0.25% FBP group compared with the other three groups, while nuclear factor erythroid 2-related factor 2 (*Nrf2*) expression was downregulated (*p* < 0.05) in the 0.5% FBP group compared with the control group. Regression analysis revealed that diets supplemented with FBP had significant linear and quadratic effects on the expressions of *CAT* (*p* = 0.042, linear model: Y = 0.984 − 0.217X), *GPX1* (*p* = 0.013, quadratic model: Y = 1.086 + 1.095X − 1.279X^2^), Kelch-like ECH-associated protein 1 (*Keap1*; *p* = 0.044, linear model: Y = 0.957 − 0.224X), and *Nrf2* (*p* = 0.004, quadratic model: Y = 0.998 − 0.989X + 0.826X^2^), respectively. However, diets supplemented with FBP had no significant effects (*p* > 0.05) on the expressions of *CAT*, heme oxygenase-1 (*HO-1*), and other oxidative indicators in the breast muscle of spent hens.

### 3.5. Identification of Bioactive Compound in FBP and Potential Targets Related to Meat Quality

**Figure 2:**
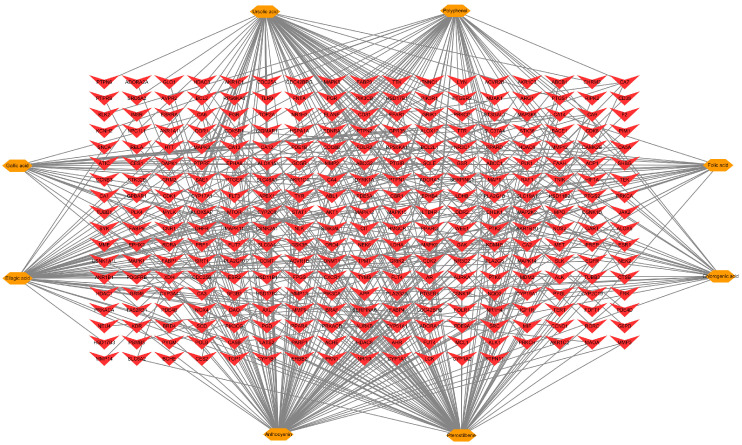
Compound-target network of fermented blueberry pomace. The orange nodes represent the bioactive compounds in fermented blueberry pomace, while the red nodes represent the targets of these bioactive compounds.

Eight bioactive compounds, including anthocyanins, polyphenols, ellagic acid, pterostilbene, folic acid, ursolic acid, chlorogenic acid, and gallic acid, were identified in FBP as postbiotics based on the literature review [21,22,23]. A total of 458 FBP targets were screened from the Swiss Target Prediction database, including 102, 53, 57, 100, 22, 75, 24, and 25 targets for anthocyanins, polyphenols, ellagic acid, pterostilbene, folic acid, ursolic acid, chlorogenic acid, and gallic acid, respectively. After removing overlapping targets, 302 targets in FBP were screened. There were 401 targets related to the meat quality of spent hens. A compound-target network of FBP comprising 302 nodes and 458 edges was constructed through Cytoscape 3.7.1 (Figure 2). A Venn diagram revealed that there were 13 common targets between FBP and meat quality (Figure S1).

**Figure 3:**
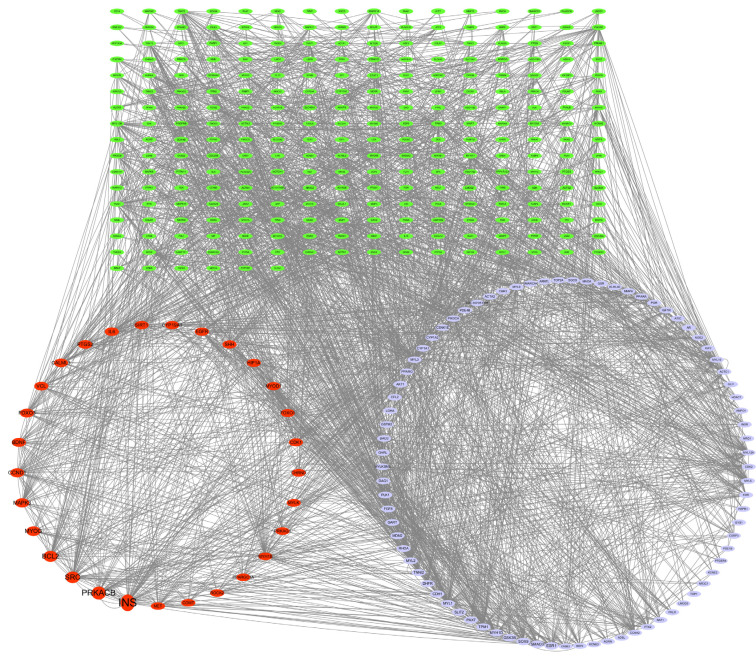
Protein–protein interaction network between core and non-core targets.

A PPI network was established using the STRING database to clarify interactions between genes and proteins (Figure S2). The TSV file from the STRING database was obtained and then imported into Cytoscape 3.7.1 to identify the core targets based on the BC value (Figure 3). The larger the nodes in the graph and the higher the BC value, the greater matching similarity, which indicated that these nodes were the core targets. As shown in Figure 3, the top four core targets included insulin (INS), protein kinase cAMP-activated catalytic subunit beta (PRKACB), steroid receptor coactivator (SRC), and B-cell lymphoma 2 (BCL2).

**Figure 4:**
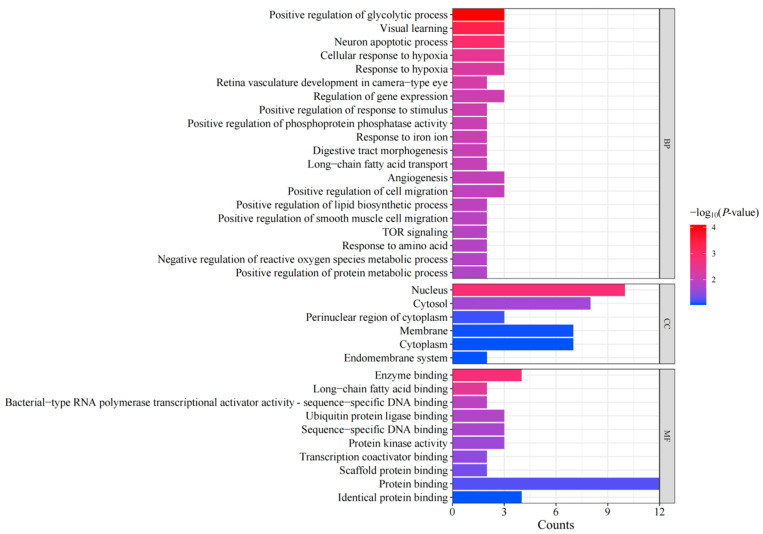
Gene Ontology pathway enrichment analysis.

A total of 65 targets in three categories, including 49 BP, 6 CC, and 10 MF, were screened through GO enrichment analysis. The top 20 enriched BP, MF, and CC terms are shown in Figure 4. The top five enriched BP terms were mainly associated with upregulation of the glycolytic process, visual learning, neuron apoptotic process, cellular response to hypoxia, and response to hypoxia. The highly enriched top five MF terms were enzyme binding, long-chain fatty acid binding, bacterial-type RNA polymerase transcriptional activator activity-sequence-specific DNA binding, ubiquitin protein ligase binding, and sequence-specific DNA binding. Additionally, CC terms included nucleus, cytosol, and perinuclear region of cytoplasm, membrane, cytoplasm, and endomembrane system.

**Figure 5:**
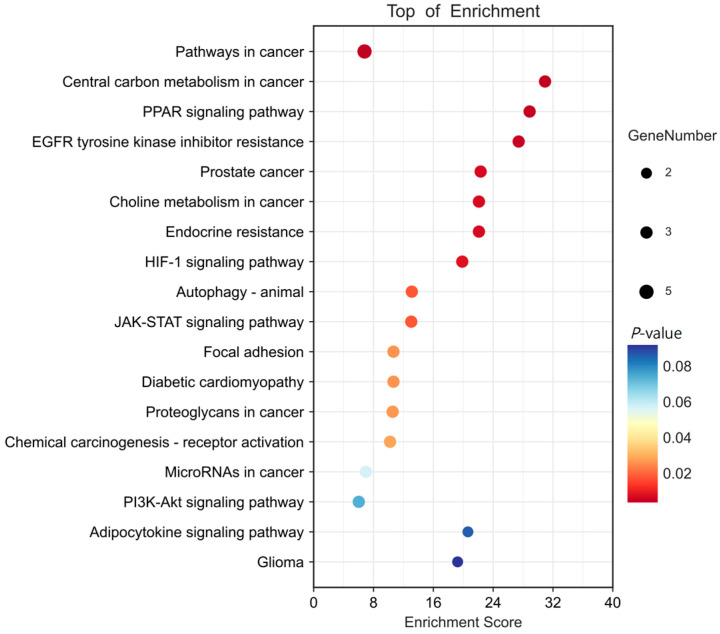
Kyoto Encyclopedia of Genes and Genomes Pathway enrichment analysis.

Furthermore, KEGG pathway enrichment analysis was carried out to investigate the signaling pathway mechanisms by which FBP improved the meat quality of spent hens. The core targets were enriched in 18 pathways, which are presented as a bubble chart (Figure 5). The highly enriched pathways were mainly involved in PI3K-Akt, JAK-STAT, HIF-1, PPAR, and other signaling pathways.

### 3.6. Molecular Docking of Bioactive Compounds

**Figure 6:**
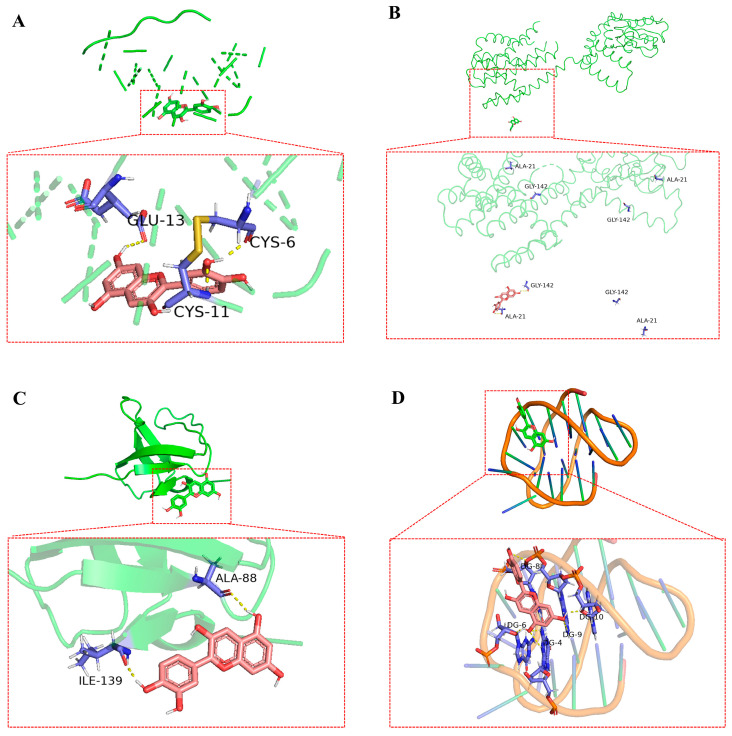
Molecular docking results of anthocyanin and core targets. (**A**) anthocyanin-INS; (**B**) anthocyanin-PRKACB; (**C**) anthocyanin-SRC; and (**D**) anthocyanin-BCL2. BCL2, B-cell lymphoma 2; INS, insulin; PRKACB, protein kinase cAMP-activated catalytic subunit beta; SRC, steroid receptor coactivator.

**Figure 7:**
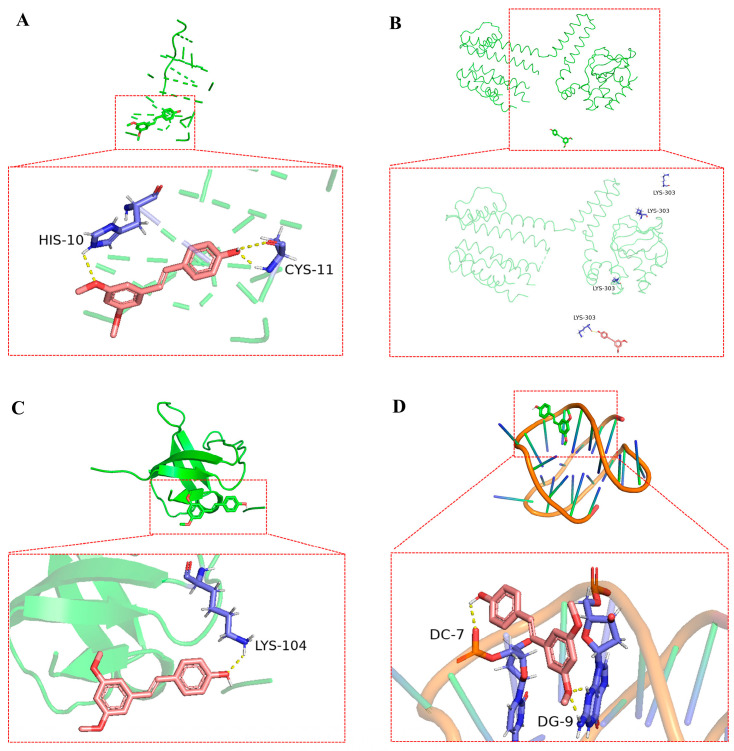
Molecular docking results of pterostilbene and core targets. (**A**) pterostilbene-INS; (**B**) pterostilbene-PRKACB; (**C**) pterostilbene-SRC; and (**D**) pterostilbene-BCL2. BCL2, B-cell lymphoma 2; INS, insulin; PRKACB, protein kinase cAMP-activated catalytic subunit beta; SRC, steroid receptor coactivator.

**Figure 8:**
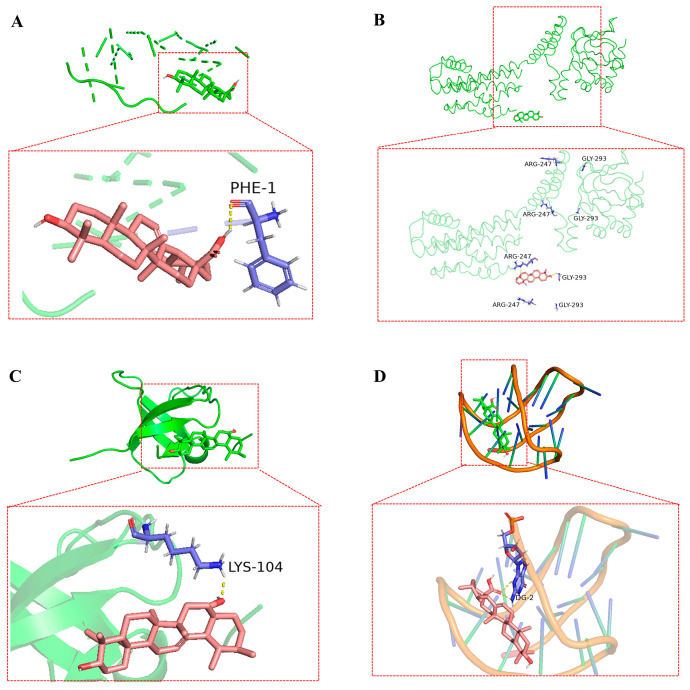
Molecular docking results of ursolic acid and core targets. (**A**) ursolic acid-INS; (**B**) ursolic acid-PRKACB; (**C**) ursolic acid-SRC; and (**D**) ursolic acid-BCL2. BCL2, B-cell lymphoma 2; INS, insulin; PRKACB, protein kinase cAMP-activated catalytic subunit beta; SRC, steroid receptor coactivator.

**Figure 9:**
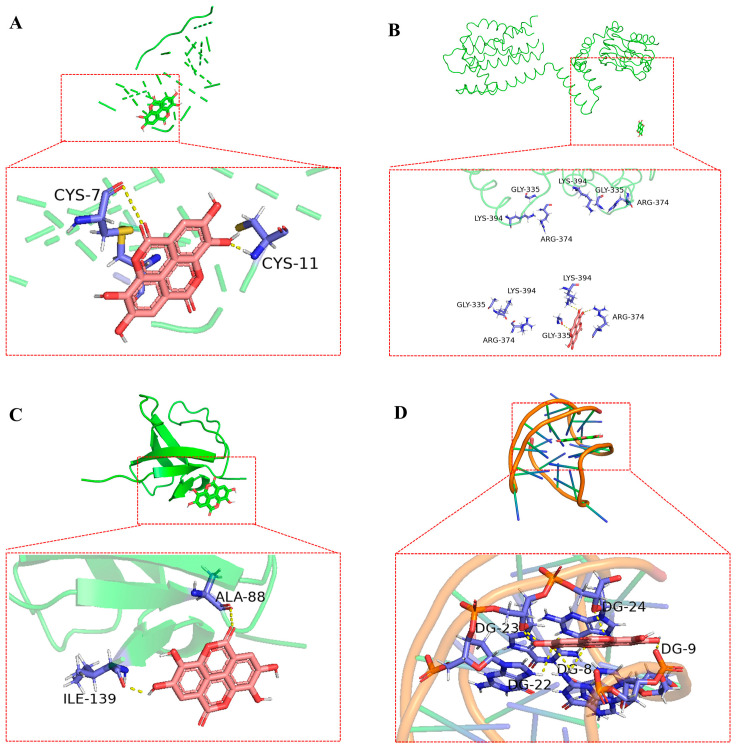
Molecular docking results of ellagic acid and core targets. (**A**) ellagic acid-INS; (**B**) ellagic acid-PRKACB; (**C**) ellagic acid-SRC; and (**D**) ellagic acid-BCL2. BCL2, B-cell lymphoma 2; INS, insulin; PRKACB, protein kinase cAMP-activated catalytic subunit beta; SRC, steroid receptor coactivator.

The top four bioactive compounds, including anthocyanins, ellagic acid, pterostilbene, and ursolic acid, were screened through network pharmacology analysis and subsequently subjected to molecular docking studies with the four core targets (including INS, PRKACB, SRC, and BCL2). The docking interactions between the receptors and ligands are visualized in Figure 6, Figure 7, Figure 8 and Figure 9. As shown in Figure 6A–D, anthocyanin established three hydrogen bonds with CYS-6, CYS-11, and GLU-13 in INS (Figure 6A); two hydrogen bonds with ALA-21 and GLY-142 in PRKACB (Figure 6B); two hydrogen bonds with ALA-88 and ILE-139 in SRC (Figure 6C); and five hydrogen bonds with DG-4, DG-6, DG-8, DG-9, and DG-10 in BCL2 (Figure 6D). Figure 7A–D demonstrates that pterostilbene formed three hydrogen bonds with HYS-10 and CYS-11 in INS (Figure 7A); one hydrogen bond with LYS-303 in PRKACB (Figure 7B); one hydrogen bond with LYS-104 in SRC (Figure 7C); and three hydrogen bonds with DG-7 and DG-9 in BCL2 (Figure 7D). Figure 8A–D demonstrates that ursolic acid interacts with PHE-1 in INS through one hydrogen bond (Figure 8A); ARG-247 and GLY-293 in PRKACB through two hydrogen bonds (Figure 8B); LYS-104 in SRC through one hydrogen bond (Figure 8C); and DG-2 in BCL2 through two hydrogen bonds (Figure 8D). Figure 9A–D indicates that ellagic acid interacted with CYS-7 and CYS-11 in INS through two hydrogen bonds (Figure 9A); GLY-335, ARG-374, and LYS-394 in PRKACB through three hydrogen bonds (Figure 9B); ALA-88 and ILE-139 in SRC through two hydrogen bonds (Figure 9C); and DG-8, DG-9, DG-22, DG-23, and DG-24 in BCL2 through eight hydrogen bonds (Figure 9D).

As presented in Table 6, all receptor–ligand combinations exhibited binding energies < 0 kJ/mol, indicating favorable binding interactions. Notably, the interaction between ursolic acid and BCL2 had the lowest binding energy (−6.39 kJ/mol). These results suggest that these four compounds contribute significant role in improving the meat quality of spent hens.

## 4. Discussion

The demand for high-quality meat is gradually increasing with the improvement of consumers’ living standards. Chicken meat is one of the major protein sources for human nutrition due to its cost-effectiveness, rapid growth, and substantial yield. Generally, spent hens are often used for deep processing, such as in the production of braised chicken, or sent to the restaurant for cooking due to their lower functional meat qualities and tougher texture. Due to these limitations, spent hens generally have lower market value and undergo minimal processing. Thus, the present study evaluated the effects of dietary FBP on carcass traits, meat quality, and antioxidant capacity of breast muscles in spent hens, leveraging network pharmacology and molecular docking approaches. Our findings indicated that diets supplemented with FBP improved meat quality by decreasing drip loss and enhancing the breast muscle’s antioxidant capacity. These improvements may be potentially linked to several bioactive compounds such as anthocyanins and ursolic acid in FBP, which exert an improving effect by targeting INS and modulating JAK-STAT and PI3K-Akt signaling pathways. These findings will provide a reference for further research into the mechanisms by which dietary FBP influences meat quality in spent hens. In practical applications, FBP is a lower-cost alternative to conventional feed. It can replace an appropriate proportion of the basal diet, thereby reducing feed costs. Furthermore, FBP contains antioxidants, such as anthocyanins and flavonoids, which can mitigate meat oxidation in laying hens. Consequently, it can be used as a functional feed additive in poultry diets.

Carcass traits, which include dressing percentage, eviscerated yield, and breast muscle percentage, are important indicators of carcass quality. A dressing percentage > 80% and an eviscerated yield > 60% are considered above the standard meat production performance [24]. In the current study, the dressing percentage of spent hens was more than 90%, and the eviscerated yield was more than 62%, indicating that the meat production performance of Yukou Jingfen spent hens exceeded the standard poultry meat production criteria. However, 0.25% FBP supplementation reduced the thigh muscle percentage, while 0.5% and 1.0% FBP had no significant effects in spent hens. The results of network pharmacology also indicated that INS was the core target. Therefore, low-dose FBP may preferentially promote glucose uptake and energy allocation in the breast muscle of laying hens by regulating the insulin signaling pathway, while the energy supply to the thigh muscles was relatively reduced, thus leading to a decreased thigh muscle ratio in the 0.25% FBP group. Conversely, Xiao et al. [25] also reported that dietary *Siraitia grosvenorii* pomace could numerically increase the percentages of breast and thigh muscles in Guangxi Ma chickens. Therefore, the observed differences in the percentage of breast muscle in the present study may be attributed to cellular variations between breast and thigh muscles, which warrants further investigations to elucidate the exact mechanism.

The liver index reflects the health status of animals to some extent. In the current study, diets supplemented with 0.5% FBP exhibited an increasing trend in the liver index of spent hens during the late laying phase, indicating partial beneficial effects of FBP on the liver index of spent hens. This finding contradicts that of Hu et al. [26], who indicated that dietary blueberry pomace supplementation decreased the liver index of broiler chickens. Bioactive compounds in blueberry pomace, such as polyphenols, can regulate lipid metabolism, thus promoting the organ index of broiler chickens. The differences between these two studies may be due to changes in the composition and content of bioactive substances before and after the fermentation of blueberry pomace. However, further studies are necessary to elucidate the underlying reason.

Meat quality is a critical factor influencing consumer acceptance of meat products. For spent layers, meat quality is influenced by various factors, including age, nutrition, health status, and stress. Meat color, which is determined by the muscular concentrations of myoglobin and hemoglobin, is a significant indicator for assessing muscle appearance. Commonly, within a specific range, the higher a* value and the lower L* and b* values indicate better meat quality. However, a previous study suggested that, in practical life, a majority of consumers exhibit a preference to purchase muscles with a pale color [27]. In the current study, diets supplemented with FBP had no significant effects on the muscle color of spent hens. Nevertheless, all FBP-supplemented groups displayed a decreasing trend in the a* value of the thigh muscle in spent hens. Polyphenols in FBP have antioxidant capacities, which may affect the oxidation state of myoglobin in muscles, leading to changes in meat color [28,29]. Although this decreasing trend in the a* value occurred for the thigh muscle value, it is not significantly conclusive; however, it aligns with consumer preferences and could enhance the marketability of the meat products.

Additionally, pH value is another important index for assessing meat quality, reflecting the post-slaughter muscle glycogen fermentation rate. The glycogen present in the muscle undergoes glycolysis, resulting in the formation of lactic acid and leading to a decrease in pH [30]. The normal pH range for fresh muscle during aging is 5.4 to 7.2 [31]. In the current study, diets supplemented with FBP (all FBP-supplemented groups, regardless of dose) decreased the pH of breast muscle at 48 h post-mortem. The short-chain fatty acids produced by intestinal microbiota may affect the glycolysis process of muscle cells by regulating energy metabolism, thereby altering the pH of muscles. Our previous study also indicated that FBP supplementation altered the composition of intestinal microbiota and metabolites in spent hens [14], which may be one of the possible reasons for the change in muscle pH. Nonetheless, the pH values for both breast and thigh muscles remained within the normal range and did not lead to excessive acidification, indicating that diets supplemented with FBP did not exert adverse effects on muscle pH nor affect or influence consumers’ purchasing decisions. Since low pH can provide a favorable environment for promoting the growth of pathogenic bacteria (such as *Pseudomonas aeruginosa*, *Escherichia coli*, etc.), it is suggested that the shelf life be extended by implementing refrigerated conditions. Collectively, these findings suggest that diets supplemented with FBP had no detrimental effects on the color or pH values of breast and thigh muscles in spent hens.

Water holding capacity (WHC) is closely linked to the tenderness, juiciness, and nutritional composition of meat and is also indirectly reflected through drip loss and cooking loss of meat. In the current study, diets supplemented with 0.5% FBP reduced the 24 h drip loss of breast muscle. In addition, supplementation with 0.25 and 0.5% FBP reduced 48 h drip loss but increased cooking loss of breast muscle, whereas 1.0% FBP had no significant effects compared to the control group. These results indicate that low-dose FBP supplementation reduces free water loss but increases bound water loss in muscle during heating. A similar previous study showed that dietary apple and cherry pomace supplementation improved meat quality by decreasing drip loss in the thigh muscle of layers [32], a finding which is consistent with the present study. The possible reason for decreasing drip loss is that the bioactive substances present in FBP can reduce the damage of harmful substances, such as free radicals, to muscles and delay muscle oxidation, thereby reducing free water loss in muscle cells [33]. Conversely, the observed increase in cooking loss may be attributed to polyphenols present in FBP, which can establish cross-links with collagen proteins. This interaction may enhance the stability of collagen fibers, reducing their thermal contraction during heating and thereby facilitating greater moisture loss [34]. However, a higher dose of FBP (1.0% FBP) had no significant effect on drip loss, a phenomenon potentially due to the presence of anti-nutritional factors introduced by high doses of FBP, which may interfere with mineral absorption and thereby indirectly influence muscle WHC [35]. Additionally, in these two studies, the reduction in drip loss was observed in different muscles (breast and thigh muscles, respectively). This discrepancy may be related to differences in nutrient substrate preferences, energy metabolism characteristics, and key regulatory pathways between breast and thigh muscles. Overall, diets supplemented with FBP reduced drip loss but increased cooking loss. However, since consumers’ perception of the juiciness of meat is also influenced by multiple factors, such as fat content, the impact of this result on final eating quality still requires further validation through sensory evaluation.

Oxidative stress impairs the structure and function of enzymes that regulate muscle sarcoplasmic calcium level, resulting in the inactivation of proteolytic enzymes associated with meat tenderization, and thus adversely affecting the meat quality of layers [36]. The concentration of MDA, the main product of lipid peroxidation, reflects the degree of injury to the cell [37]. SOD, CAT, and GSH-PX are natural antioxidant enzymes in the animal’s body that neutralize free radicals produced by oxidative stress, reflecting the antioxidant capacity of animals [38]. In the current study, diets supplemented with 0.5% FBP enhanced the T-AOC activity compared with the 0.25% FBP group, while also increasing the SOD activity compared with the control and 0.25% FBP groups. These findings indicated that 0.5% FBP improved the antioxidant capacity of breast muscle compared to 0.25% FBP and control groups by enhancing the activities of antioxidant enzymes, thereby improving the meat quality of spent hens, without any significant effects from the higher dose of FBP (1.0%). Borges et al. [39] found that anthocyanins in blueberries have high antioxidant activity using an online high-performance liquid chromatography antioxidant detector system. Therefore, it is speculated that the enhancement of antioxidant capacity in the breast muscles of spent hens in the current study may be related to the antioxidant bioactive substances present in FBP, such as anthocyanins. At higher concentrations, the anthocyanins and other polyphenols present in FBP act as potential antioxidants. However, when supplemented with medium dosages of FBP (0.5%), due to a moderate redox potential, a small amount of ROS is produced via the Fenton reaction in specific environments, particularly where metal ions like iron and copper are present [40]. While this ROS level is too low to cause significant damage, it is sufficient to partially stimulate lipid peroxidation. This process elevates the MDA level, which may be the reason for the increased MDA concentration observed in the 0.5% FBP group.

Through the literature review, the main bioactive compounds of FBP were identified as anthocyanins, polyphenols, ellagic acid, pterostilbene, folic acid, ursolic acid, chlorogenic acid, and gallic acid [21,22,23]. For example, Salzano et al. [41] found that diets supplemented with red orange and lemon extract, both rich in anthocyanins, effectively improved the meat quality of Saanen suckling kids by increasing the content of unsaturated fatty acids while decreasing saturated fatty acids, atherosclerosis, and thrombosis indexes. Similarly, another study also reported that dietary anthocyanin-rich corn cobs improved the meat quality of lambs, particularly by enhancing the aroma in cooked boneless loin chops [42]. Dietary supplementation with 0.04% tea polyphenols has been shown to improve the meat quality of broiler chickens, as evidenced by increasing a* value and pH_24h_, along with reducing the shear force of breast muscle [43]. Moreover, Li et al. [44] reported that dietary 16 mg/kg folic acid supplementation increased pre-slaughter weight, dressing and meat percentages, and a* value of the meat in ewes. Overall, bioactive compounds present in FBP, including anthocyanins, polyphenols, and folic acid, can potentially improve the meat quality of spent hens.

To explore the core targets of FBP in enhancing meat quality, a PPI network was constructed. The results of the PPI network analysis identified INS, PRKACB, SRC, BCL2, MYOG, MAPK3, CCND1, and BDNF as the core targets associated with the improvement of meat quality. BCL2 protein, an anti-apoptotic protein, can regulate cell apoptosis by modulating intracellular Ca^2+^ flux and activating upstream caspase-9 and downstream caspase-3 [45,46]. The degree of apoptosis is closely correlated to meat quality; specifically, a higher degree of apoptosis is associated with an increased degradation rate of muscle fibers, leading to reduced water retention and tenderness of the meat. *MYOG*, a member of the MRFs family (including *MYOD*, *MYOG*, *MYF5*, and *MRF4*) of myogenic regulatory factors, plays a vital role in myoblast fusion and muscle fiber formation [47,48]. The number and size of muscle cells and fibers determine the quality of meat. Therefore, we postulated that the *MYOG* gene might be one of the key genes influencing the terminal differentiation of muscle cells and, thus, the meat quality. These findings suggest that FBP may enhance meat quality by targeting the key genes such as *INS*, *MYOG*, and *IL-6* through bioactive compounds (such as anthocyanins). Lower binding energy indicates stronger ligand-receptor affinity, leading to a more stable structure. In the current study, the binding energy in all combinations was <0 kJ/mol, confirming the binding activity between bioactive compounds and core targets.

The GO and KEGG pathway enrichment analyses were performed to investigate the mechanism by which FBP enhances the meat quality of spent hens. The GO analysis showed that FBP was involved in multiple biological functions, including interactions with the nucleus, cytosol, and other cellular components. These functions included the positive regulation of the glycolysis process, the regulation of gene expression, and cell migration. Consequently, FBP exerts molecular functions such as enzyme binding, long-chain fatty acid binding, and ubiquitin protein ligase binding, ultimately improving the meat quality of spent hens.

The KEGG pathway enrichment analysis revealed that the target genes for FBP in improving meat quality were mainly associated with several signaling pathways, including pathways in cancer, PPAR, HIF-1, JAK-STAT, and PI3K-Akt signaling pathways, highlighting the multi-target mechanism of bioactive compounds in FBP. For example, Zanders et al. [49] found that IL-6 upregulates the *SOCS3* expression by exerting its effects on muscle cells through a complex of glycoprotein 130 and the IL-6 receptor, thereby activating the JAK-STAT signaling pathway. SOCS3 downregulates atrophy-related genes and reduces protein degradation by inhibiting the INS/PI3K/Akt signaling pathway, thereby enhancing protein synthesis and promoting muscle growth [49]. These findings suggest that FBP, through its bioactive compounds, modulates core targets such as INS and IL-6, regulating signaling pathways, including the JAK-STAT and PI3K-Akt pathways. This mechanism likely contributes to beneficial effects such as muscle growth regulation and improved meat quality of spent hens.

However, the application of FBP in poultry diets presents several challenges. First, its nutritional composition, such as fiber and polyphenol content, varies significantly with the blueberry variety, juice extraction process, and storage conditions. This variability complicates the formulation of consistent and balanced diets, hindering standardized production. Second, beneficial probiotics and active enzymes produced during fermentation can viability during storage, particularly under high temperatures, posing a practical challenge for maintaining the product’s bioactivity until it is fed. Moreover, this study was limited to a 56-day trial with laying hens and a small experimental scale compared to commercial operations. Given the potential impacts of high-density breeding and environmental stressors in commercial settings on the efficacy of FBP, long-term and large-scale trials are necessary to further validate its stability and economic viability.

## 5. Conclusions

In summary, dietary FBP supplementation significantly improved breast muscle quality of spent hens, particularly by reducing the drip loss after post-slaughter and enhancing the antioxidant capacity through increasing T-AOC and SOD activities of breast muscle without adversely affecting the carcass traits. Notably, among different doses of FBP, 0.5% FBP demonstrated better beneficial effects than other treatments. However, FBP supplementation reduced the pH of breast muscle at 48 h post-mortem while increasing cooking loss. These changes were within normal ranges for meat quality and consumer acceptance. Additionally, network pharmacology and molecular docking analyses identified anthocyanin, ursolic acid, and ellagic acid as the primary bioactive compounds in FBP, targeting key proteins such as INS, PRKACB, SRC, and BCL2. These core targets may activate the PI3K-AKT and JAK-STAT signaling pathways, thereby contributing to improved meat quality in spent hens.

## Figures and Tables

**Table 1:** The composition and nutrient levels of the basal diet (%, as feed basis).

Composition	Content	Nutrient	Level ^2^
Corn	64.20	Crude protein	15.56
Soybean meal	21.60	Crude fat	5.10
Soybean oil	1.20	Metabolizable energy, MJ/kg	11.38
Limestone	8.00	Lysine	0.95
Premix ^1^	5.00	Methionine	0.33
Total	100.00	Calcium	3.52
		Total phosphorus	0.42

^1^ Premix provided per kg of diet: Vitamin A, 10,000 IU; vitamin D_3_, 3000 IU; vitamin E, 20 IU; vitamin K_3_, 1.75 mg; vitamin B_1_, 2 mg; vitamin B_2_, 6 mg; vitamin B_6_, 3 mg; pantothenic acid, 8.5 mg; vitamin B_12_, 0.02 mg; nicotinamide, 40 mg; folic acid, 1 mg; biotin, 0.24 mg; choline chloride, 450 mg; methionine, 1.3 g; lysine, 0.95 g; calcium, 5 g; phosphorus, 0.75 g; copper, 8 mg; iron, 75 mg; manganese, 100 mg; zinc, 65 mg; iodine, 0.8 mg; selenium, 0.3 mg; sodium chloride, 3 g. ^2^ The nutrient levels are measured value in triplicate, and metabolizable energy is a calculated value followed by NRC.

**Table 2:** The primer sequences used for RT-qPCR.

Gene Names	Accession No.	Primer Sequence (5′–3′)	Product Size (bp)
*CAT*	NM_001031215.2	F: AGATGGCGTATGACCCTAGC R: CCTCTGATAATTGGCCACGC	173
*GPX1*	NM_001277853.2	F: ATGTTCGAGAAGTGCGAGGT R: AGTTCCAGGAGACGTCGTTG	160
*HO-1*	NM_205344.1	F: ATGCCTACACCCGCTATTTG R: ATCTCAAGGGCATTCATTCG	178
*Keap1*	MN416132.1	F: CATCAACTGGGTGCAGTACG R: AGGGTGAGGTCCTGGAAGAT	183
*NQO1*	NM_001277619.1	F: AAGAAGATTGAAGCGGCTGA R: GCATGGCTTTCTTCTTCTGG	166
*Nrf2*	NM_205117.1	F: CCACCCTAAAGCTCCATTCA R: ATTCTTGCCTCTCCTGCGTA	217
*SOD1*	NM_205064.1	F: ATTACCGGCTTGTCTGATGG R: CCTCCCTTTGCAGTCACATT	173
*SOD2*	NM_204211.2	F: CCTTCGCAAACTTCAAGGAG R: CCAGCAATGGAATGAGACCT	162
*β-actin*	NM_205518.1	F: ATGAAGCCCAGAGCAAAAGA R: GGGGTGTTGAAGGTCTCAAA	223

Abbreviations: *CAT*, catalase; *GPX1*, glutathione peroxidase 1; *HO-1*, heme oxygenase-1; *Keap1*, Kelch-like ECH-associated protein 1; *NQO1*, NAD(P)H quinone dehydrogenase 1; *Nrf2*, nuclear factor erythroid 2-related factor 2; *SOD*, superoxide dismutase.

**Table 3:** Effects of FBP on carcass trait and organ index of spent hens.

Items	Treatments				SEM	*p*-Values		
	Control	0.25% FBP	0.5% FBP	1.0% FBP		FBP	Linear	Quadratic
Live weight, kg	1.72	1.68	1.72	1.74	0.028	0.877	0.620	0.668
Dressed weight, kg	1.59	1.55	1.59	1.60	0.026	0.931	0.729	0.813
Dressing percentage, %	92.24	92.61	92.67	91.81	0.278	0.693	0.495	0.316
Percentage of half-eviscerated yield, %	79.43	78.52	79.14	77.85	0.442	0.610	0.256	0.895
Percentage of eviscerated yield, %	64.57	63.66	62.81	63.74	0.496	0.665	0.589	0.295
Percentage of breast muscle, %	11.96	10.56	12.37	12.04	0.265	0.067	0.409	0.605
Percentage of thigh muscle, %	13.39 ^a^	11.16 ^b^	12.60 ^ab^	12.81 ^ab^	0.274	0.017	0.924	0.059
Liver index, g/kg	17.38	17.94	20.62	19.83	0.676	0.077	0.095	0.426
Spleen index, g/kg	1.08	1.05	1.18	1.03	0.029	0.594	0.688	0.202
Abdominal fat index, g/kg	42.89	42.06	34.14	34.88	2.398	0.720	0.167	0.609

Abbreviation: FBP, fermented blueberry pomace; SEM, standard error of the mean. Data are expressed as means with their SEM (*n* = 8). ^a–b^ Mean values within a row with different superscript letters are significantly different (*p* < 0.05).

**Table 4:** Effects of FBP on meat quality of spent hens.

Items	Treatments				SEM	*p*-Values		
	Control	0.25% FBP	0.5% FBP	1.0% FBP		FBP	Linear	Quadratic
Breast muscle								
L*	53.40	52.85	53.91	54.11	0.308	0.322	0.241	0.790
a*	11.27	11.25	11.34	11.30	0.240	0.999	0.940	0.958
b*	12.49	12.92	12.75	12.95	0.250	0.919	0.606	0.800
pH_45min_	6.12	6.10	5.95	5.98	0.074	0.832	0.455	0.715
pH_48h_	5.94 ^a^	5.65 ^b^	5.73 ^b^	5.72 ^b^	0.028	0.001	0.050	0.006
24 h drip loss, %	6.75 ^a^	6.48 ^a^	2.85 ^b^	3.91 ^ab^	0.565	0.017	0.028	0.130
48 h drip loss, %	8.43 ^a^	5.13 ^b^	4.54 ^b^	5.47 ^ab^	0.475	0.007	0.044	0.006
Cooking loss, %	14.58 ^b^	17.79 ^a^	17.42 ^a^	15.47 ^ab^	0.437	0.009	0.914	0.003
Shear force, N	26.12	25.17	27.40	29.78	1.169	0.921	0.186	0.652
Thigh muscle								
L*	40.61	41.92	42.32	40.70	2.605	0.494	0.895	0.123
a*	19.37	17.30	18.14	17.82	1.689	0.079	0.209	0.146
b*	7.86	8.12	8.19	8.55	1.342	0.804	0.317	0.971
pH_45min_	6.63	6.52	6.51	6.48	0.153	0.223	0.081	0.308
pH_48h_	5.93	6.00	5.92	5.96	0.118	0.529	0.946	0.862
24 h drip loss, %	4.46	5.97	5.38	4.87	2.610	0.773	0.947	0.355
48 h drip loss, %	6.25	8.65	6.78	5.39	3.009	0.245	0.307	0.204
Cooking loss, %	32.57	32.10	33.36	32.67	1.654	0.552	0.671	0.658

Abbreviations: FBP, fermented blueberry pomace; L*, brightness; a*, redness; b*, yellowness; SEM, standard error of the mean. Data are expressed as means with their SEM (*n* = 8). ^a–b^ Mean values within a row with different superscript letters are significantly different (*p* < 0.05).

**Table 5:** Effects of FBP on the antioxidant capacity in the breast muscle of spent hens.

Items	Treatments				SEM	*p*-Values		
	Control	0.25% FBP	0.5% FBP	1.0% FBP		FBP	Linear	Quadratic
GSH, U/mgprot	0.80	0.88	0.93	0.84	0.035	0.834	0.769	0.179
GSH-PX, U/mgprot	4.14	4.57	6.91	6.15	0.540	0.118	0.128	0.299
MDA, nmol/mgprot	0.27 ^b^	0.29 ^ab^	0.48 ^a^	0.27 ^b^	0.029	0.018	0.889	0.015
SOD, U/mL	7.86 ^b^	7.22 ^b^	13.92 ^a^	10.27 ^ab^	0.852	0.009	0.144	0.103
T-AOC, mmol/mg	0.07 ^ab^	0.05 ^b^	0.11 ^a^	0.09 ^ab^	0.006	0.005	0.104	0.352

Abbreviations: FBP, fermented blueberry pomace; SEM, standard error of the mean; GSH, glutathione; GSH-PX, glutathione peroxidase; MDA, malondialdehyde; SOD, superoxide dismutase; T-AOC, total antioxidant capacity. Data are expressed as means with their SEM (*n* = 8). ^a–b^ Mean values within a row with different superscript letters are significantly different (*p* < 0.05).

**Table 6:** The binding energy level of bioactive compounds and core targets.

Ingredient	Targets	Binding Energy, kJ/mol
Anthocyanin	INS	−4.32
	PRKACB	−2.27
	SRC	−2.82
	BCL2	−5.10
Ursolic acid	INS	−6.28
	PRKACB	−4.57
	SRC	−4.89
	BCL2	−6.39
Pterostilbene	INS	−5.29
	PRKACB	−2.69
	SRC	−2.92
	BCL2	−3.98
Ellagic acid	INS	−5.08
	PRKACB	−2.76
	SRC	−3.61
	BCL2	−5.00

Abbreviations: INS, insulin; PRKACB, protein kinase cAMP-activated catalytic subunit beta; SRC, steroid receptor coactivator; BCL2, B-cell lymphoma 2.

## Data Availability

The datasets generated and/or analyzed during the current study are available from the corresponding authors upon reasonable request.

## Supplementary Materials

The following supporting information can be downloaded at https://www.mdpi.com/article/10.3390/ani15192799/s1, Figure S1: Venn diagram of the targets of fermented blueberry pomace and the targets of meat quality; Figure S2: The process of topological screening for the PPI network.
