# Supplementary material for: Beneficial Effects of Fermented Blueberry Pomace Supplementation on Carcass Traits, Meat Quality, and Antioxidant Capacity of Spent Hens
Source: Animals (Basel). 2025 Sep 25;15(19):2799. doi: 10.3390/ani15192799 (PMC12524072; doi:10.3390/ani15192799)
Supplement: Supplementary file 1 [file animals-15-02799-s001.zip › animals-3834715-supplementary.pdf]

Supplementary Figures

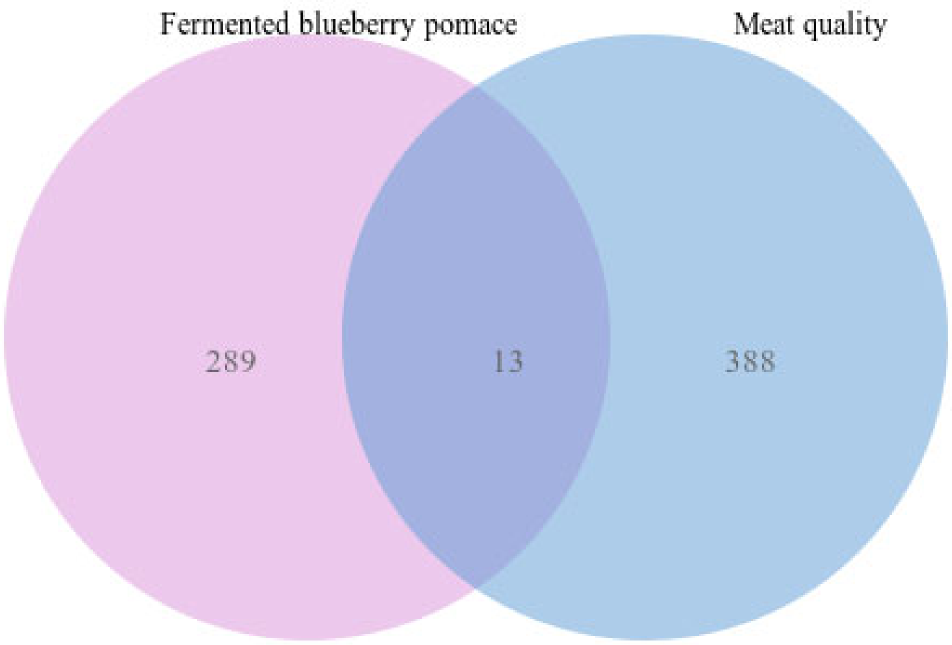

Figure S1. Venn diagram of the targets of fermented blueberry pomace and the targets of meat quality

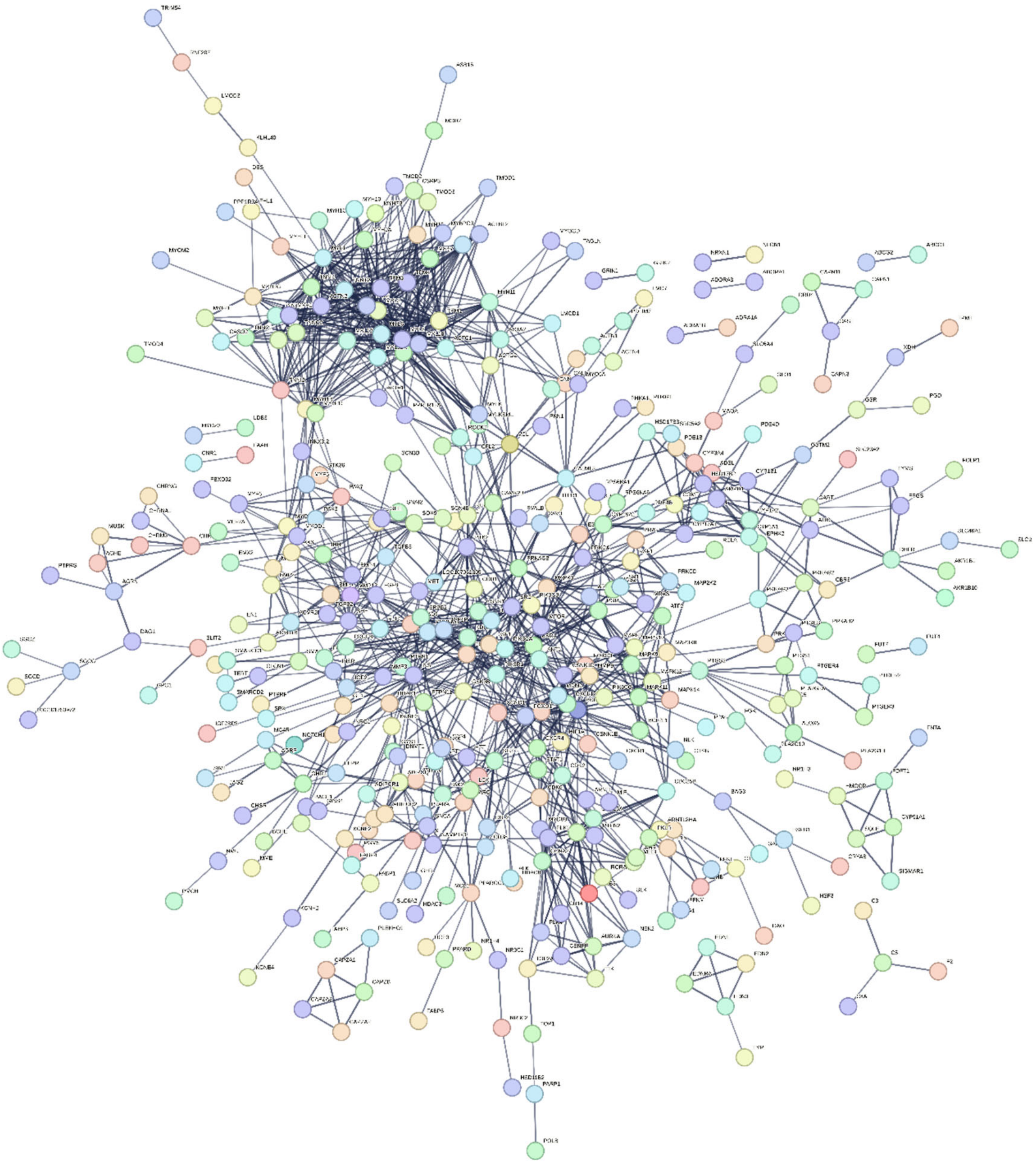

Figure S2. The process of topological screening for the PPI network
